# Code-based Syndromic Surveillance for Influenzalike Illness by International Classification of Diseases, Ninth Revision

**DOI:** 10.3201/eid1302.060557

**Published:** 2007-02

**Authors:** Nicola Marsden-Haug, Virginia B. Foster, Philip L. Gould, Eugene Elbert, Hailiang Wang, Julie A. Pavlin

**Affiliations:** * Walter Reed Army Institute of Research, Silver Spring, Maryland, USA; † Air Force Institute for Operational Health, Brooks City Base, Texas, USA; 1 Current affiliation: Northwest Center for Public Health Practice, Seattle, Washington, USA; 2 Current affiliation: US Census Bureau, Suitland, Maryland, USA; 3 Current affiliation: Uniformed Services University of the Health Sciences, Bethesda, Maryland, USA

**Keywords:** Disease outbreaks, Surveillance, Influenza, ICD-9 codes, research

## Abstract

With the spread of avian influenza, use of automated data streams to rapidly detect and track human influenza cases has increased. We performed correlation analyses to determine whether International Classification of Diseases, Ninth Revision (ICD-9), groupings used to detect influenzalike illness (ILI) within an automated syndromic system correlate with respiratory virus laboratory test results in the same population (r = 0.71 or 0.86, depending on group). We used temporal and signal-to-noise analysis to identify 2 subsets of ICD-9 codes that most accurately represent ILI trends, compared nationwide sentinel ILI surveillance data from the Centers for Disease Control and Prevention with the automated data (r = 0.97), and found the most sensitive set of ICD-9 codes for respiratory illness surveillance. Our results demonstrate a method for selecting the best group of ICD-9 codes to assist system developers and health officials who are interpreting similar data for daily public health activities.

## Abstract 2

ICD-9 codes collected automatically in a syndromic system are sensitive and specific in detecting outbreaks caused by respiratory viruses.

Inevitable annual cycles of influenza and other respiratory pathogens pose a significant threat to work and productivity (*1*–*3*). Epidemics can have dramatic economic and medical ramifications, such as the influenza pandemic of 1918 (*4*,*5*). During the last few years we have witnessed the emergence of severe acute respiratory syndrome (SARS) and new pathogenic avian influenza strains. These events have brought respiratory illnesses to the attention of the general public; most recently, the highly publicized potential for pandemic influenza due to recombinant influenza strains has generated tremendous public anxiety. Moreover, lingering fears about influenzalike illness (ILI) symptoms related to bioterrorism have further accentuated the need for improved early detection of respiratory disease outbreaks.

This atmosphere of concern motivated an intense effort to develop new surveillance methods (*6*). Public health officials are now augmenting traditional disease surveillance, e.g., laboratory-based methods, with nontraditional analysis of electronic medical records for more timely monitoring of infectious disease patterns. The Centers for Disease Control and Prevention (CDC), along with many health departments, universities, and government organizations, has participated in research and development of syndromic surveillance systems. Some of these systems have been designed for local surveillance in a single metropolitan area, while others cover broad geographic areas, including multiple jurisdictions (*7*,*8*).

Since 2001, the Department of Defense (DOD) has been using the Electronic Surveillance System for the Early Notification of Community-based Epidemics (ESSENCE) for syndromic surveillance of active duty military and their beneficiaries (*9*,*10*). This system captures patient ambulatory data coded according to the International Classification of Diseases, Ninth Revision (ICD-9), from all permanent military treatment facilities (MTFs) that treat active duty personnel, retirees, and their family members worldwide. It provides a large amount of data for surveillance, with >300,000 average weekly outpatient visits to primary care and emergency facilities for any reason. The system automatically performs daily analysis of visits classified in each of 8 syndrome groups, such as respiratory and gastrointestinal illnesses and fever.

Military basic training sites have historically experienced frequent respiratory epidemics among troops in crowded housing (*11*–*14*), and active surveillance for ILI is conducted year-round. To improve early detection of such epidemics and in response to pandemic and bioterrorism concerns, an automated ILI surveillance report was also incorporated into ESSENCE in 2002 (*9*).

Critics of syndromic surveillance have voiced apprehension about the use of nontraditional data and the ability of these systems to detect outbreaks (*15*–*17*). Skepticism about ICD-9 data in particular revolves around whether data coded at the time of visit accurately reflects true illness, given the potential for coding of nonspecific symptoms and unconfirmed diagnoses and for provider or coder variations in code selection (*18*). We sought to evaluate the effectiveness of using ESSENCE as an early detection system for ILI and to determine the most parsimonious set of ICD-9 codes to use for ILI surveillance. We compared the ICD-9–based ILI data in ESSENCE to data from the laboratory-based DOD Global Influenza Surveillance Program and the sentinel reports from CDC’s US Influenza Sentinel Providers Surveillance Network. We compared diagnostic codes from ESSENCE both individually and as a group to the volume of positive respiratory specimens and weekly sentinel reports. Through trend, correlation, and signal-to-noise analysis, we identified a subset of diagnostic codes that best corresponds with influenza patterns.

## Methods

### ESSENCE Data Collection

ESSENCE captures outpatient visit data recorded as ICD-9 codes at or shortly after the patient encounter (*10*). A central, secure-link electronic database allows for daily submission of data, although reporting time from the MTFs averages from 1 to 4 days. Data entry practices vary by location, but each MTF is set up to batch-send data to the central database on a daily basis; in most locations, 80% of all ICD-9 codes are received within 4 days. The ESSENCE server collects de-identified data from the central database every 8 hours; at each time of collection, ESSENCE is refreshed with newly submitted data from MTFs. With each cycle, data are grouped by ICD-9 codes, recounted, and republished into syndromes, including ILI. Most syndromes are published as daily counts, but the ILI syndrome is grouped as weekly data. The published data for the ILI syndrome is also updated and republished every 8 hours, but the initial publication of the weekly data does not occur until a full week (running Sunday to Saturday) is completed.

We created our original ILI syndrome group by reviewing the ICD-9 code and listing and choosing those that could represent potential ILI cases. According to this classification, visits are counted as ILI if their diagnostic code is either fever, an included acute respiratory code, or unspecified viral illness. The 29 codes in the original ILI group are listed in Table 1. Each week ESSENCE calculates the percentage of visits for ILI among the total number of outpatient primary care and emergency department visits.

**Table 1:** Original set of 29 ICD-9 codes included in the influenzalike illness syndrome in ESSENCE*

ICD-9 code	Description	Specificity and severity rank†
079.89	Viral infection NEC*	4
079.99	Viral infection NOS*	4
460	Nasopharyngitis, acute	4
462	Pharyngitis, acute	4
464.00	Laryngitis, acute, without obstruction	4
464.10	Tracheitis, acute, without obstruction	4
464.20	Laryngotracheitis, acute without obstruction	4
465.0	Laryngopharyngitis, acute	4
465.8	Infectious upper respiratory, multiple sites, acute NEC	4
465.9	Infectious upper respiratory, multiple sites, acute NOS	4
466.0	Bronchitis, acute	3
466.11	Bronchiolitis due to respiratory syncytial virus	3
466.19	Bronchiolitis, acute, due to other infectious organism	3
478.9	Disease, upper respiratory NEC/NOS	4
480.0	Pneumonia due to adenovirus	2
480.1	Pneumonia due to respiratory syncytial virus	2
480.2	Pneumonia due to parainfluenza	2
480.8	Pneumonia due to virus NEC	2
480.9	Viral pneumonia unspecified	2
484.8	Pneumonia in other infectious disease NEC	2
485	Bronchopneumonia, organism NOS	2
486	Pneumonia, organism NOS	2
487.0	Influenza with pneumonia	1
487.1	Influenza with respiratory manifestation NEC	1
487.8	Influenza with manifestation NEC	1
490	Bronchitis NOS	3
780.6	Fever	4
784.1	Pain, throat	4
786.2	Cough	4

*ICD-9, International Classification of Diseases, Ninth Revision; ESSENCE, Electronic Surveillance System for the Early Notification of Community-based Epidemics; NOS, not otherwise specified; NEC, not elsewhere classified. †Specificity and severity rank: 1, most severe or specific; 4, least severe or specific.

### Direct Comparison of Respiratory Specimens Matched to Outpatient Visits

The DOD Influenza Surveillance program, located at the Air Force Institute for Operational Health at Brooks Air Force City-Base, Texas, collects specimens and screens for a variety of viral respiratory pathogens, including influenza A and B, respiratory syncytial virus, adenovirus, and herpes simplex virus (*19*,*20*). All MTFs are encouraged to submit specimens on a year-round basis, but sentinel sites are specifically directed to submit 6–10 specimens per week during the official influenza season, week 40 in the first year through week 20 in the second year (generally October through early May). The program guidelines state that specimens should only be obtained from patients meeting a clinical case definition of ILI, which at the time of this study was a fever ≥100.5°F (38°C) and either a cough or sore throat (*20*).

We matched individual specimens with outpatient clinic visits that occurred within a 5-day range around the date of specimen collection by using a unique patient code that links the records but does not identify the patient. This analysis included encounters for active duty personnel, dependents, and retirees during the 2-year period of June 2002 to June 2004, but was limited to visits to US Air Force MTFs because we had the ability to link laboratory and outpatient encounter records at these locations. Specimens were first matched to a visit that occurred on the same day that the specimen was collected; those specimens that matched were excluded from subsequent match attempts. Remaining specimens were then sequentially matched to visits 1 day earlier, 1 day later, 2 days earlier, and 2 days later than the date listed as date collected. Upon each iteration of this process, specimens were excluded from the remaining potential match pool if successfully matched to a visit. The purpose of this window approach is to obtain as many matches as possible and allow for some discrepancy between the visit date and the date of collection.

For each encounter linked to a specimen, we selected a single ICD-9 code per individual specimen. Some specimens had more than 1 encounter on the day matched, so we used the following algorithm for selection of the ICD-9 code: if 1 of the ICD-9 codes present was from the ILI syndrome list, it was selected. In cases in which patients had multiple ILI diagnoses, the more specific (for influenza first and other diseases second) or severe code was used, e.g., if both pneumonia and throat pain were included, pneumonia was selected; if pneumonia and influenza with pneumonia were included, influenza with pneumonia was selected (Table 1). If no ILI codes were used for the visit, the code closest to an infectious respiratory diagnosis was used; we gave priority to infectious disease or respiratory codes first, to general symptoms second, to other diagnoses third, and “V codes” (supplementary classification of factors influencing health status and contact with health services) last. We then measured the frequency of positive viral specimens by ICD-9 code.

### Trend Analysis of Unmatched Syndromic ICD-9 Codes and DOD Influenza Specimens

A second analysis compared DOD-wide positive specimens from the DOD Global Influenza Surveillance Program to ICD-9 data without matching from October 2000 through December 2004. We extended the date range for this analysis because more data were available for the DOD-wide population. We compared the trend of the DOD-wide specimens to the trends of each individual ICD-9 code in the ILI set, as well as additional codes frequently used in association with the collection of viral specimens in the matched Air Force analysis. We selected individual codes that had trends similar to that of the specimens and evaluated trends for groupings of 3–10 ICD-9 codes. We then measured the association between individual and grouped ILI codes with the positive viral specimens through both standard and lagged correlation analysis. We calculated lagged correlation coefficients by shifting the ICD-9 data by three 1-week increments both forward and backward, while holding the positive specimens constant.

We also performed signal-to-noise analysis of individual codes. First, we defined the influenza season as weeks in which the weekly count of positive specimens was greater than the mean of positive specimens for the study period. We then calculated means and standard deviations of the daily counts for each ICD-9 code. We defined signal as the mean during the influenza season minus the mean during the noninfluenza period and noise as the standard error during the noninfluenza period. The ratio of signal-to-noise evaluated whether individual codes would provide a good signal during the influenza season.

We used 4 separate criteria to select the best performing ICD-9 codes: individual code trend; high correlation coefficient (>0.6 preferable); high signal-to-noise ratio (≥1.5 preferable); and a substantial percentage of positive specimens for either all pathogens (>35%) or influenza virus (>20%). Codes fitting these specifications were retained for further analysis. Because the signal from codes used less often might be lost when combined with more frequently used codes, we created 2 new groupings, 1 with high-volume codes (ILI-large) and 1 with low-volume codes (ILI-small). We defined high-volume codes as being used >50× per day on average or >75,000× during the 4-year study period.

### Assessment of Daily Algorithm Performance on ICD-9 Data

We performed another analysis to assess the utility of running daily statistical algorithms on the ESSENCE ILI group, in a way similar to algorithms run on the other 8 syndrome groups. ILI is currently reported as a weekly percentage of visits without statistical alerts. Outbreak detection in ESSENCE is based on a mixed time-series model that combines regression and exponentially weighted moving average (EWMA) algorithms (*10*,*21*,*22*). The number of patient visits is related not only to the previous day’s count but also to specific day of the week. The model treats holidays and weekends differently from the days following them. It reduces, or smoothes, artificial peaks in the data, which result not from true epidemics but from surges in patient visits after clinic closures, so that these peaks do not cause frequent false alarms. Likewise, the model accounts for fewer persons seeking care on weekends or during holidays, so these fluctuations do not affect the predictions. For this analysis, we ran the mixed EWMA and regression model on daily counts of the original ESSENCE ILI group, as well as on counts of the new ILI-large and ILI-small groups.

### Weekly ILI Trend Comparison between CDC Sentinel Surveillance and DOD ICD-9 Data

From October through May, providers within the US Influenza Sentinel Providers Surveillance Network submit weekly reports to CDC of the total number of patients seen and the number of those patients with ILI (*23*). CDC calculates and reports weekly percentages by region. In this system, ILI is defined as a “fever (temperature of ≥100°F (37.8°) plus either a cough or a sore throat, in the absence a known cause other than influenza.” To confirm the results we found in our comparison of DOD surveillance systems, we analyzed the trends and correlation between weekly DOD-wide ESSENCE ILI groupings and nationwide CDC data during 3 influenza seasons: 2001–02, 2002–03, and 2003–04.

### Statistical Analysis

We used Stata version 8.0 (Stata Corporation, College Station, TX, USA) and SAS versions 8.2 and 9 (SAS Institute, Cary, NC, USA) for the direct comparison of specimen data and patient visits and SAS versions 8.2 and 9 for statistical modeling and analysis. The ESSENCE-mixed EWMA and regression models were designed by using SAS macros. This research protocol was approved by the Institutional Review Board at the Walter Reed Army Institute of Research.

## Results

During the study period, 7,389 Air Force specimens were taken for the matched analysis. We found an ICD-9–coded visit within the 5-day window surrounding the sample collection date for 6,236 (84.4%), with most of those specimens matching on the exact day (5,267, 84.5%). Of the 6,236 specimens with a match, 339 patients (5.4%) had >1 visit recorded: 321 had 2 visits, 12 had 3 visits, and 1 patient had 4 visits for the same day. Tables 2 and 3 show a breakdown of how the match worked, including multiple visits and multiple ICD-9 codes per visit. We gave preference to the highest order diagnosis for 68 patients who had multiple ILI diagnoses. For the 96 patients who had multiple visits without an ILI code, we selected the closest diagnosis to an infectious disease or 1 depicting respiratory symptoms.

**Table 2:** Data showing match of Air Force respiratory virus specimens to ICD-9 coded visits, January 2002–July 2003*

Match day	No. specimens†
Did not match (removed from study)	1,153
Clinic day – 2 = specimen day	47
Clinic day – 1 = specimen day	125
Exact day match	5,267
Clinic day + 1 = specimen day	680
Clinic day + 2 = specimen day	117
Total	7,389

*ICD-9, International Classification of Diseases, Ninth Revision. †No. specimens obtained by day of matching visit.

**Table 3:** Data showing match of Air Force respiratory virus specimens to ICD-9 coded visits, January 2002–July 2003*

No. visits/type	No. specimens
2 visits recorded (n = 321)	
Both non-ILI	90
1 ILI; 1 non-ILI	164
Both ILI	67
3 visits recorded (n = 12)	
3 non-ILI	6
1 ILI; 2 non-ILI	5
2 ILI; 1 non-ILI	1
4 visits recorded (n = 1)	
1 ILI; 3 non-ILI	1

*ICD-9, International Classification of Diseases, Ninth Revision; ILI, influenza-like illness. †No. specimens obtained by day of matching visit.

Tables 4 and 5 show the number of specimens associated with each ICD-9 code, as well as the percentage of those specimens that tested positive for any viral respiratory pathogen and for influenza virus. We found many of the ILI codes to either be infrequently used with a viral specimen or to have a low percentage of positive specimens. Four codes not in the original ILI group (otitis media, acute suppurative otitis media, acute sinusitis, and acute tonsillitis) were frequently used with the collection of viral specimens.

**Table 4:** Laboratory specimens matched with outpatient visit data, Air Force data, June 2001–June 2003*†

ICD-9 code	Description	No.	% Positive for any viral respiratory pathogen	% Positive for influenza A or B
079.99	Viral infection NOS*	783	51	40
780.6	Fever	611	74	13
466.0	Bronchitis, acute	146	39	15
486	Pneumonia, organism NOS	238	40	12
465.9	Infectious upper respiratory, multiple sites, acute NOS	1,251	61	20
461.9	Acute sinusitis, unspecified	66	47	28
382.9	Otitis media NOS	51	31	27
460	Nasopharyngitis, acute	286	36	23
490	Bronchitis NOS	26	39	26
786.2	Cough	52	37	23
487.1	Influenza with respiratory manifestation NEC	372	54	49
487.8	Influenza with manifestation NEC	46	46	43
487.0	Influenza with pneumonia	4	100	75
465.8	Infectious upper respiratory, multiple sites, acute NEC	38	68	28
466.11	Bronchiolitis due to respiratory syncytial virus	33	61	9
466.19	Bronchiolitis, acute, due to other infectious organism	88	30	3
480.2	Pneumonia due to parainfluenza	0	NA	NA
462	Pharyngitis, acute	637	40	13
480.1	Pneumonia due to respiratory syncytial virus	2	0	0
382.00	Otitis media, acute suppurative NOS	30	47	30
480.9	Viral pneumonia unspecified	5	40	40
478.9	Disease, upper respiratory NEC/NOS	1	100	0
461.8	Other acute sinusitis	0	NA	NA
465.0	Laryngopharyngitis, acute	3	67	0
484.8	Pneumonia in other infectious disease NEC	1	0	0
480.8	Pneumonia due to virus NEC	6	50	16
079.89	Viral infection, NEC	33	36	33
485	Bronchopneumonia, organism NOS	0	NA	NA
464.20	Laryngotracheitis, acute without obstruction	0	NA	NA
463	Acute tonsillitis	57	46	0
784.1	Pain, throat	14	14	0
464.10	Tracheitis, acute, without obstruction	1	0	0
480.0	Pneumonia due to adenovirus	0	NA	NA
464.00	Laryngitis, acute, without obstruction	2	0	0

*International Classification of Diseases, Ninth Revision (ICD-9); NOS, not otherwise specified; NEC, not elsewhere classified (as listed in the ICD-9); NA, not .assessed. †Laboratory specimen data matched with outpatient visit data in Air Force analysis, June 2001–June 2003.

**Table 5:** Unmatched outpatient visit data and final influenzalike illness (ILI) syndromic groupings*

ICD-9 code	Unmatched data†					ILI group	
	Total volume of code use (2001–04)	Average daily count	Correlation with positive specimens	p value	Signal -to-noise ratio	Original	New (final)
079.99	1,115,143	718	0.7746	<0.0001	2.84	Yes	Large
780.6	470,770	303	0.7545	<0.0001	2.55	Yes	Large
466.0	632,256	407	0.6693	<0.0001	2.17	Yes	Large
486	322,397	208	0.7180	<0.0001	2.09	Yes	Large
465.9	3,989,688	2,569	0.6758	<0.0001	1.91	Yes	Large
461.9	741,085	477	0.6017	<0.0001	1.81	No	Large
382.9	1,185,809	764	0.6286	<0.0001	1.76	No	Large
460	361,139	233	0.5552	<0.0001	1.55	Yes	Large
490	297,918	192	0.6337	<0.0001	1.50	Yes	Large
786.2	545,510	351	0.5573	<0.0001	1.24	Yes	Large
487.1	62,340	40	0.8696	<0.0001	5.59	Yes	Small
487.8	8,973	6	0.7926	<0.0001	4.74	Yes	Small
487.0	5,093	3	0.6205	<0.0001	3.11	Yes	Small
465.8	72,042	46	0.6384	<0.0001	1.86	Yes	Small
466.11	18,377	12	0.4800	<0.0001	1.83	Yes	–
466.19	68,127	44	0.5257	<0.0001	1.65	Yes	–
480.2	451	0	0.3316	<0.0001	1.64	Yes	–
462	1,436,325	925	0.5468	<0.0001	1.61	Yes	–
480.1	1,790	1	0.4083	<0.0001	1.58	Yes	–
382.00	277,270	179	0.4868	<0.0001	1.58	No	–
480.9	10,852	7	0.4562	<0.0001	1.44	Yes	–
478.9	7,434	5	0.4296	<0.0001	1.23	Yes	–
461.8	123,913	80	0.3083	<0.0001	1.14	No	–
465.0	33,760	22	0.4804	<0.0001	1.11	Yes	–
484.8	4,312	3	0.3202	<0.0001	1.11	Yes	–
480.8	11,708	8	0.3501	<0.0001	1.07	Yes	–
079.89	17,729	11	0.3355	<0.0001	1.05	Yes	–
485	7,954	5	0.4180	<0.0001	0.99	Yes	–
464.20	3,539	2	0.3852	<0.0001	0.88	Yes	–
463	168,499	108	0.3176	<0.0001	0.08	No	–
784.1	59,516	38	0.2994	<0.0001	0.59	Yes	–
464.10	1,736	1	0.2560	0.0002	0.56	Yes	–
480.0	287	0	0.0889	0.2082	0.24	Yes	–
464.00	22,470	14	0.1133	0.1085	0.14	Yes	–

*NOS, not otherwise specified; NEC, not elsewhere classified (as listed in the International Classification of Diseases, 9th Revision). ‡ Department of Defense–wide outpatient visit data, October 2000–December 2004.

For the unmatched DOD-wide analysis, we found 15,914 samples taken during the study period, of which 6,340 (39.8%) were positive for any viral respiratory pathogen, and 2,210 (13.9%) were positive for influenza A or B. Temporal analysis showed that as a group, the original ILI syndrome follows the same seasonal pattern as that for positive specimens. Individual ICD-9 code trends for influenza, fever, unspecified viral infection, otitis media, and upper respiratory infection (multiple sites) correlated well with those of the positive specimens (Tables 3, 4). Codes that did not correlate with positive specimen trends included acute tonsillitis and throat pain.

Many individual codes that correlated well with the positive specimens also tended to have high signal-to-noise ratios (Table 5). Moreover, the percentage of positive specimens associated with many of these codes also tended to be high. Based on the results of these 3 tests and their individual trends, we selected 14 ICD-9 codes for ILI surveillance. We used the frequency of individual code use during the 4-year analysis period to group 10 of the 14 codes into the ILI-large group and the other 4 into the ILI-small group, as indicated in Tables 4 and 5.

**Figure 1:**
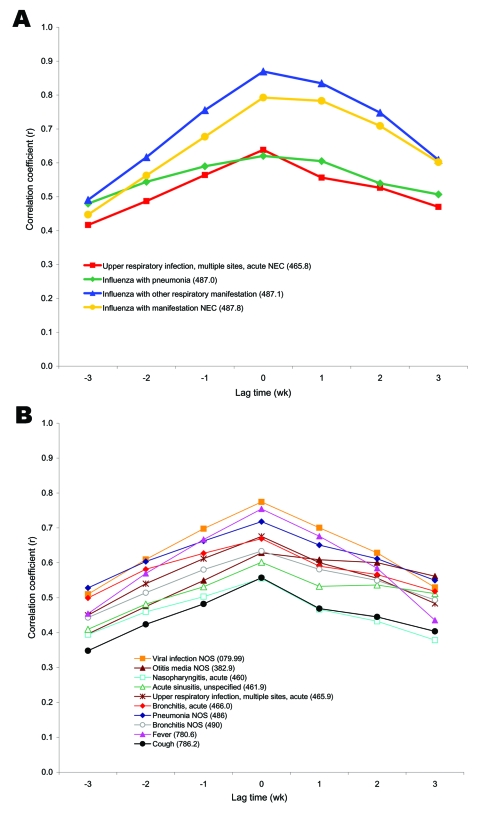
Lagged correlation analysis between individual codes from the International Classification of Diseases, Ninth Revision (ICD-9), and unmatched positive respiratory specimens from October 2000 to December 2004. Each of the individual ICD-9 codes that had high correlation and signal-to-noise ratio when compared with positive influenza laboratory specimens taken during the same time frame (Table 5) were compiled into new large and small influenzalike illness (ILI) groups (large codes were used >50×/day on average) and compared again to the positive specimens through lagged analysis. The ICD-9 data were shifted by three 1-week increments both forward and backward, while holding the positive specimens constant. A) Lag time correlation coefficients for ICD-9 codes in the new large count ILI group. B) Lag time correlation coefficients for ICD-9 codes in the new Small count ILI group.NEC, necrotizing enterocolitis; NOS, not otherwise specified.

Lagged correlation analysis found that the codes of both subsets tend to peak at the same time as the number of positive specimens (Figure 1). However, the ILI-Small group codes, while still peaking centrally, tended to have curves slightly skewed to the right in the lagged correlation plot, indicating that they may be more likely to follow, rather than predict, the increases in ILI visits.

**Figure 2:**
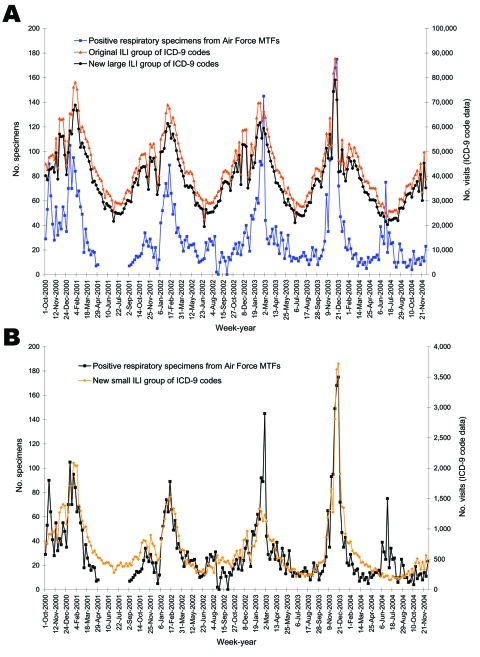
Weekly trends among unmatched visits coded by International Classification of Diseases, Ninth Revision (ICD-9), and specimens positive for any viral respiratory pathogen from October 2000 to December 2004. Based on correlation to positive cultures and signal-to-noise ratios, new large and small influenzalike illness (ILI) categories were created. The number of positive specimens is depicted on the left y-axis and compared to the number of visits for the original, new large and new small ILI ICD-9 categories, as shown in the right y-axis. A) Original ILI and new ILI-large groups with positive specimens; B) new ILI-small group with positive specimens. MTFs, military treatment facilities; CI, confidence interval.

After establishing the new small and large ILI groups, we found that the weekly temporal trends closely follow those of positive respiratory specimens (Figure 2). Correlation coefficients of the weekly data were 0.72 (p<0.0001), 0.71 (p<0.0001), and 0.86 (p<0.0001) for the original, ILI-large, and ILI-small groups, respectively.

**Figure 3:**
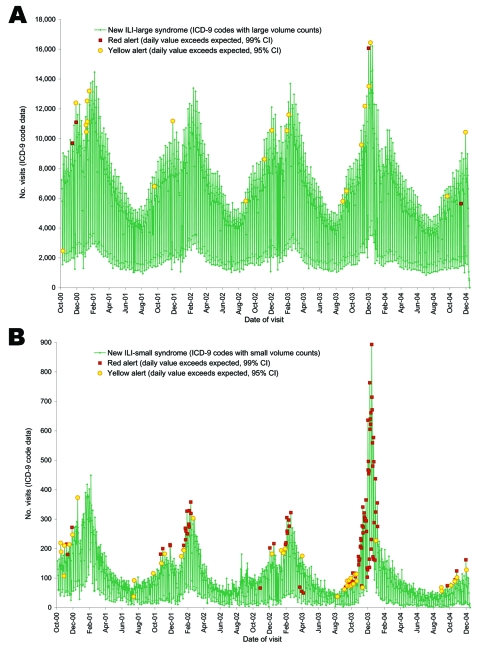
Newly created groups of International Classification of Diseases, Ninth Revision (ICD-9), codes for influenzalike illness (ILI) based on correlation to positive cultures and signal-to-noise ratios were run with anomaly detection algorithms. Two groups, large and small, were created with ICD-9 codes that had an average use of >50× per day in the large group with the remainder in the small group. Daily counts of the codes in the Large and Small syndromic groups were plotted from October 2000 to December 2004. An algorithm based on a mixed time series model that combines regression and exponentially weighted moving average (EWMA) is used to detect potential outbreaks and takes into account weekends and holidays. Yellow alerts occur when the daily value exceeds that expected with a 95% confidence interval, and red alerts occur when the amount exceeds the expected with a 99% confidence interval. A) Large syndrome group. B) Small syndrome group.

We ran the EWMA/regressive model on 4 years of daily DOD outpatient data in each of the 3 comparison groups (Figure 3). Multiple seasonal outbreaks of respiratory illness were identified with alerts for all groupings. The daily algorithm triggered alerts much more frequently on the ILI-small group than on the large group; the algorithm for the small grouping tended to be more responsive to smaller fluctuations in the data.

**Figure 4:**
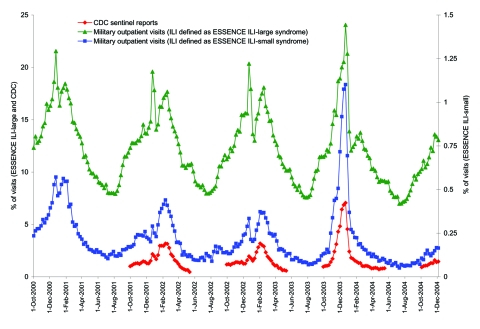
Percentage of visits for influenza-like illness (ILI) using both the large and small syndrome groups among military outpatient visits nationwide compared with Centers for Disease Control and Prevention (CDC) sentinel clinician reports from October 2001 through December 2004. Data are grouped weekly from Sunday through Saturday. CDC data are only obtained during the influenza season. ESSENCE, Electronic Surveillance System for the Early Notification of Community-based Epidemics.

Direct comparison of the nationwide US Influenza Sentinel Providers Surveillance Network with the ESSENCE ILI groupings showed very similar trends during each of the previous 3 seasons (Figure 4). Further analysis showed that CDC data were very strongly correlated with data from the ILI-small group; with correlation coefficients 0.97 (p<0.0001), 0.87 (p<0.0001), and 0.99 (p<0.0001) for the 2001–02, 2002–03, and 2003–04 seasons, respectively. Correlation coefficients for the ILI-large group were also very strong, although not quite as high: 0.88 (p<0.0001), 0.77 (p<0.0001), and 0.93 (p<0.0001), respectively.

## Discussion

In our experience with ESSENCE, the ILI surveillance report has been 1 of the most useful components. Military public health officials, and now some civilian health departments, use ESSENCE to monitor the ILI grouping for early signs of the influenza season and other common febrile respiratory outbreaks. In a similar manner, CDC now monitors ILI by using the same DOD data within the BioSense system. This study shows that the DOD outpatient ICD-9 data are indeed useful and accurate for routine influenza surveillance.

Critical analysis of the ICD-9 codes within ESSENCE ILI group showed that approximately half of the codes were associated with specimens positive for respiratory pathogens, including influenza. Temporal trends confirmed that most codes followed the same trends over time as positive specimens. Codes with low correlation to positive specimens and different temporal trends have been removed from the group to produce more parsimonious groups. The less-specific ILI-large group may be more useful for the initial detection of influenza season and for detecting other respiratory illnesses that initially cause similar symptoms, whereas the ILI-small group is more specific but also more likely to signal slightly later than the large group because providers should use these codes cautiously until influenza cases have been confirmed. However, both groupings have been shown to be useful indictors of an impending influenza season.

ESSENCE should produce reports of ILI activity faster than both the laboratory-based DOD Global Influenza Surveillance Program and the CDC sentinel ILI system because it is able to collect and analyze data more rapidly than specimens and provider reports can be processed. The weekly data are reported in ESSENCE immediately on completion of a full week, whereas the DOD laboratory data have an inherent lag time because of the time required for specimen shipping, laboratory testing, analysis, and reporting. The CDC sentinel reporting system similarly lags behind because of the passive nature of data collection and additional time required to compile and post results. The automated data collection also allows for the potential to analyze data more frequently than the current weekly standard. Our analysis successfully identified seasonal outbreaks by using a combination algorithm on daily data, based on aggregated data for a given day. The algorithm runs every 8 hours (more or less frequently depending on administrator settings) and recalculates on the basis of newly received data. Daily detection algorithms can be instituted on the large and small groups simultaneously to best detect ILI outbreaks.

The results of this study support previous findings on the ability of automated systems to capture the same trends as traditional surveillance. The Minnesota Department of Health found that an ILI grouping of ICD-9 data from a health maintenance organization in the Minneapolis–St. Paul area correlated with reported deaths from pneumonia and influenza (*24*). Ambulatory ICD-9 codes were also successfully used for surveillance of respiratory illnesses in Massachusetts and were highly correlated with hospital admissions that had a lower proportion of discharged patients with a diagnosis of respiratory illness (*25*). Our study also supports evidence that using nontraditional electronic data for syndromic surveillance may enable health providers to recognize and detect the influenza season faster than with traditional means. In a similar study of nontraditional data, the New York City Department of Health and Mental Hygiene reported that their syndromic system, based on chief complaints at emergency departments, detected the first citywide signs of influenza activity sooner than laboratory- and sentinel-based surveillance (*26*).

We have established that ICD-9–based surveillance that uses the ILI-large and ILI-small groups is an effective tool for influenza surveillance. We suggest that health agencies use these syndrome groups as a model for developing similar systems. However, we strongly emphasize that developers perform critical analysis of the individual codes collected in their data and carefully consider not only the clinical basis for code inclusion but also which diagnoses are more likely to cause background “noise” rather than contribute to the signal. Our own evaluation illustrates the importance of such critical review, as we found that both throat pain and acute tonsillitis had more noise than signal. Asthma and chest pain are included in other syndromic systems (*24*); however, in the DOD data, these tend to occur year-round with fairly high volume and contribute more noise than signal in the DOD ambulatory data. Studies of systems that use such broad categories for ILI surveillance have yielded lower correlation of ICD-9 data with mortality and laboratory-based data (*24*). Data sources differ dramatically in population coverage, quality and accuracy, and most important, in their ability to reflect true disease patterns. Our method for defining and assessing syndrome groupings for ICD-9–based surveillance should assist developers in parsing, analyzing, and interpreting their own data.
